# Supplementary material for: An oligoclonal antibody durably overcomes resistance of lung cancer to third‐generation EGFR inhibitors
Source: EMBO Mol Med. 2017 Dec 6;10(2):294–308. doi: 10.15252/emmm.201708076 (PMC5801506; doi:10.15252/emmm.201708076)
Supplement: Supplementary file 1 — Appendix [file EMMM-10-294-s001.pdf]

APPENDIX

Table of content:

- **Appendix Table S1**
- **Appendix Table S2**
- **Appendix Table S3**
- **Appendix Table S4**
- **Appendix Table S5**
- **Appendix Supplementary Methods**

Appendix Table S1:
Absolute counts obtained using the digital arrays (12.765; see Fig. EV5b and Fig. EV5c).

Assay	DNA amount	Sample Name	FAM-MGB Est. Targets	FAM-MGB Est.T-95L	FAM-MGB Est.T-95U	VIC-MGB Est. Targets	VIC-MGB Est.T-95L	VIC-MGB Est.T-95U
del746/750	2.5 ng	PC9ER	450	402	499	45	31	58
		PC9ER-AZDR	770	700	843	11	4	17
T790M	2.5 ng	PC9ER	190	160	218	376	333	419
		PC9ER-AZDR	663	600	727	398	353	443
C797S	2.5 ng	PC9ER	1	0	2	544	489	600
		PC9ER-AZDR	224	192	255	470	420	520
RNASE P	2.5 ng	PC9ER	0	0	0	151	125	176
		PC9ER-AZDR	0	0	0	118	95	139

Appendix Table S2:
Absolute counts obtained using the 12.765 digital arrays (see Figure 6D and Fig. EV5f).

Dilution	Total DNA amount	Sample name	C797S-FAM Est. Targets	C797S-FAM Est.T-95L	C797S-FAM Est.T-95U	WT-VIC Est. Targets	WT-VIC Est.T-95L	WT-VIC Est.T-95U
(1:1)	10 ng	PC9ER	0	0	0	2544	2292	2875
		PC9ER-AZDR	1238	1133	1353	2602	2341	2949
(1:2)	5 ng	PC9ER	0	0	0	1204	1102	1315
		PC9ER-AZDR	687	622	753	1480	1354	1620
(1:4)	2.5 ng	PC9ER	0	0	0	632	572	695
		PC9ER-AZDR	340	299	380	773	702	846
(1:8)	1.25 ng	PC9ER	0	0	0	338	297	378
		PC9ER-AZDR	187	158	215	368	325	411
(1:16)	0.625 ng	PC9ER	0	0	0	163	136	189
		PC9ER-AZDR	94	73	112	175	147	201
	1 pg	CTRL-WT	0	0	0	5104	4230	6965
		CTRL-MUT	5104	4230	6965	0	0	0

Appendix Table S3:
List of real-time PCR primers used in the study.

Target	Use	Forward Primer (5'-3')	Reverse Primer (5'-3')
MICA	real-time PCR	TAAAATCCGGCGTAGTCCTG	GCATGTCACGGTAATGTTC
IL-8	real-time PCR	GTCTGCTAGCCAGGATCCAC	GCTTCCACATGTCCTCACAA
ULBP1	real-time PCR	GCTTCTGCACCTGCTGTCT	AGGCCTTGAACTTCACACCA
ULBP2	real-time PCR	CAGAGCAACTGCGTGACATT	AACTGCCAAGATCCACTGCT
EGFR	real-time PCR	AGGACCAAGCAACATGGTCA	CCTTGCAGCTGTTTTCACCT
B2M	real-time PCR	GGCATTCCTGAAGCTGAC	TCTTTGGAGTACGCTGGATAG

Appendix Table S4:
List of primers used for genotyping assays and for digital PCR.

Target Mutation	Use	Primers (for/rev)	Probes
EGFR del746/750	genotyping /dPCR	TGGATCCCAGAAGGTGAGAAA	VIC-TATCAAGGAATTAAGAGAGGC -MGB-NFQ
		GCTTCATCGAGGATTTCCTTGT	FAM-TCGCTATCAAAACACT -MGB-NFQ
EGFR T790M	genotyping /dPCR	GCCTGCTGGGCATCTG	VIC-ATGAGCTGCGTGATGAG-MGB-NFQ
		TCTTTGTGTTCCCGGACATAGTC	FAM-ATGAGCTGCATGATGAG-MGB-NFQ
EGFR C797S	genotyping /dPCR	GCCTGCTGGGCATCTG	VIC-TTCGGCTGCCTCCTG-MGB-NFQ
		TCTTTGTGTTCCCGGACATAGTC	FAM-TTCGGCAGCCTCC-MGB-NFQ

Appendix Table S5:
List of exact *p*-values and additional statistical informations

Figure	Samples additional info		n	technical repetitions	Stars	Exact p value	Statistical test	Comparison test
1E		Vehicle vs 3xmAbs	5	6-8 slides/tumor	***	0.00033759	one-way ANOVA	Tukey
		Vehicle vs Osimertinib			****	0.00000774	one-way ANOVA	Tukey
2E	PC9ER	Vehicle vs 3xmAbs	3	8 slides/tumor	*	0.04105734	one-way ANOVA	Tukey
		Vehicle vs Osimertinib			****	0.00000245	one-way ANOVA	Tukey
	H1975	Vehicle vs Osimertinib			***	0.00022189	one-way ANOVA	Tukey
3D		Vehicle vs 3xmAbs	4	8 slides/tumor	****	0.000000024	one-way ANOVA	Tukey
3E		Vehicle vs 3xmAbs	4	100-200 cells each/condition	**	0.0019841	one-way ANOVA	Tukey
		Vehicle vs Osimertinib			***	0.0003206	one-way ANOVA	Tukey
3F		Vehicle vs 3xmAbs	4	100-200 cells each/condition	**	0.00177182	one-way ANOVA	Tukey
		Vehicle vs Osimertinib			****	0.00000025	one-way ANOVA	Tukey
3G		Vehicle vs 3xmAbs	4	100-200 cells each/condition	**	0.00193779	one-way ANOVA	Tukey
3H	γH2AX	Vehicle vs 3xmAbs	5	6-8 slides/tumor	**	0.0031973	one-way ANOVA	Tukey
		Vehicle vs Osimertinib			****	0.000097	one-way ANOVA	Tukey
	p16	Vehicle vs 3xmAbs			***	0.0008426	one-way ANOVA	Tukey
4D		Vehicle vs 3xmAbs	6	7-8 slides/tumor	**	0.001371046	one-way ANOVA	Tukey
		Vehicle vs Osimertinib			***	0.00056159	one-way ANOVA	Tukey
		Vehicle vs 3xmAbs+Osimertinib			****	0.000000046	one-way ANOVA	Tukey
		Osimertinib vs 3xmAbs+Osimertinib			*	0.043247098	one-way ANOVA	Tukey
5C	EGFR	Vehicle vs Osimertinib	3	3 slides/tumor	**	0.00129461	one-way ANOVA	Tukey
		Vehicle vs 3xmAbs			****	0.00000148	one-way ANOVA	Tukey
		Vehicle vs 3xmAbs+Osimertinib			****	0.000000013	one-way ANOVA	Tukey
		3xmAbs vs 3xmAbs+Osimertinib			***	0.00064323	one-way ANOVA	Tukey
	HER2	Vehicle vs Erlotinib			*	0.02852016	one-way ANOVA	Tukey
		Vehicle vs Osimertinib			****	0.00000629	one-way ANOVA	Tukey
		Vehicle vs 3xmAbs			****	0.00000044	one-way ANOVA	Tukey
		Vehicle vs 3xmAbs+Osimertinib			****	0.000000011	one-way ANOVA	Tukey
		Osimertinib vs 3xmAbs+Osimertinib			****	0.000000001	one-way ANOVA	Tukey
	HER3	Vehicle vs Osimertinib			***	0.00089902	one-way ANOVA	Tukey
		Vehicle vs 3xmAbs			****	0.00007014	one-way ANOVA	Tukey
		Vehicle vs 3xmAbs+Osimertinib			****	0.00001517	one-way ANOVA	Tukey
		Osimertinib vs 3xmAbs+Osimertinib			****	0.00000133	one-way ANOVA	Tukey
F6C		PBS vs 3xmAbs	3	3	**	0.006027	T-test	
F6F		Vehicle vs 3xmAbs	3	5-6 slides/tumor	***	0.00015	one-way ANOVA	Tukey
EV1a	PC9ER	PBS vs CTX+TRZ+33	3	3	**	0.00340709	one-way ANOVA	Tukey
	H1975	PBS vs. CTX+TRZ			**	0.00127656	one-way ANOVA	Tukey
		PBS vs. CTX+TRZ+33			***	0.0006543	one-way ANOVA	Tukey
EV3f	PC9ER	ULBP2	3	3	*	0.0435	T-test	
	H1975	ULPB1			*	0.02961	T-test	
		IL-8			*	0.0386154	T-test	
EV3h	no ptretreatment	PBS vs 3xmAbs	3	3	**	0.00260444	one-way ANOVA	Bonferroni
	pretreatment	PBS vs CTX			*	0.01982778	one-way ANOVA	Bonferroni
		PBS vs 3xmAbs			***	0.00021116	one-way ANOVA	Bonferroni
EV3c		Vehicle vs 3xmAbs	3	6-8 slides/tumor	*	0.013745	one-way ANOVA	Tukey
		Vehicle vs Osimertinib			***	0.000606	one-way ANOVA	Tukey
EV3d		Vehicle vs 3xmAbs	3	6-8 slides/tumor	***	0.00064764	one-way ANOVA	Tukey

Appendix Supplementary Methods

Materials

Murine antibodies to HER3 and other receptors were generated in our laboratory. Cetuximab and trastuzumab were obtained from BMS and Roche, respectively. For immunoblotting, we used antibodies specific to p65 (#sc-372, 1:1000), ERK2 (#sc-271458, 1:1000), GAPDH (#sc-25778, 1:1000) and 53BP1 (#sc-22760, 1:1000), all fromfrom Santa Cruz Biotechnology. Anti-tubulin (#T6199, 1:5000), anti-GAPDH (1:1000) and anti-phosphorylated ERK (#M9692, 1:10000) were from

Sigma-Aldrich. Anti-EGFR (#ALX-804-064-C100, 1:1000) was from Alexis. Antibodies to phosphorylated EGFR (Tyr1068; #2234S, 1:1000), pAKT (Ser473; #4060S, 1:1000), AKT (#2938, 1:1000), caspase 3 (#9665, 1:1000), cleaved caspase 3 (#9664, 1:1000), BIM (#2933, 1:1000), caspase 9 (#9508, 1:1000), PARP (#9542, 1:1000), cleaved PARP (#5625, 1:1000), and KIP/p27 (#3686, 1:1000) were obtained from Cell Signaling. KI67-specific (#Ab16667, 1:1000), p16 (#Ab108349, 1:1000) and γH2AX (#16667, 1:1000) antibodies were from AbCam. DCR2 (#ADI-APP-371-E, 1:1000), p15 (#C0287-1, 1:1000) and p21 (#556431, 1:1000) antibodies were respectively from Enzo Life Bioscience, Assay Biotech and BD Bioscience. For flow cytometry we used antibodies to EGFR (clone AY13), HER2 (clone 24D2) and HER3 (clone 1B4C3) that were respectively conjugated with the following fluorophores: Alexa Fluor 488, allophycocyanin, and phycoerythrin (BioLegend Inc.). Erlotinib and afatinib were obtained from LC Laboratories, and AZD9291 and CO-1686 were obtained from Selleckchem. 3-(4,5-dimethylthiazol-2-yl)-2,5-diphenyl tetrazolium bromide (MTT) was obtained from Sigma-Aldrich. Nuclear Fast Red solution was purchased from Sigma.

Cell cultures

Unless indicated, cells were obtained from the American Type Tissue Culture Collection (ATCC). The PC9 set of cell lines included the erlotinib-sensitive PC9 (EGFR del746–750) and erlotinib-resistant PC9ER (del746–750+T790M) NSCLC cell lines. The PC9ER-AZDR derivative cells we generated were grown under the same conditions as the parental cell lines, namely in RPMI-1640 medium supplemented with 10% fetal calf serum (Life Technologies) and antibiotics. NL20 cells (normal human lung epithelial cells), also from ATCC, were used as control. All cell lines were routinely checked for mycoplasma contamination.

Cell growth, apoptosis and immunoblotting assays

Apoptosis was determined using flow cytometry. Annexin V, 7-amino-actinomycin D (7-AAD) and a kit were from BioLegend. For immunoblotting, cells were extracted in Lysis Buffer (50 mM Hepes (pH 7.5), 10% glycerol, 150 mM NaCl, 1% Triton X-100, 1 mM EDTA, 1 mM EGTA, 10 mM NaF, 0.1 mM Na_3VO_4, and a complete protease inhibitor cocktail). Tumor xenografts were processed with Lyisng Matrix D Tubes (MP Biomedicals) prior to protein extraction. Following protein separation using gel electrophoresis and transfer to nitrocellulose membranes, immunoblotting was performed according to the antibody manufacturers' recommendation. Antibody binding to membrane blots was detected using horseradish peroxidase–secondary antibodies (Jackson ImmunoResearch Laboratories), followed by treatment with ECL Western blotting detection reagents (from GE Healthcare).

Immunoblotting analyses

Whole cell extracts were resolved using gel electrophoresis and proteins were transferred to nitrocellulose membranes. The membranes were blocked with 5% non-fat dried milk in Tris-buffered saline containing 0.05% Tween-20 (TBS-T), and incubated overnight with the desired primary antibody. Subsequently, the membranes were washed three times, and bound antibodies were detected using the appropriate horseradish peroxidase-conjugated secondary antibodies (for 60 minutes),

followed by probing using an ECL PlusWestern blotting Detection System (GE Healthcare Bio-Sciences). ECL was detected using a Molecular ImagerR ChemiDoc™ System (from Bio-Rad Laboratories) and images were acquired using the ImageLab Software.

Cell cycle analysis

Following drug treatment, cells were incubated for 60 minutes with BrdU (10 μM), washed, harvested and fixed in 70% ethanol (at 4°C). Prior to analysis, cells were incubated in a denaturation solution (2N HCl, 0.5% Triton-X100; 30 min), followed by a neutralization solution (0.1 M sodium borate, pH 8.5; 30 min). BrdU was probed using an anti-BrdU antibody coupled to APC. Total cellular DNA was stained in a propidium iodide (PI) solution supplemented with RNase A. This was followed by data acquisition using BD FACS Aria III. Cell cycle distribution was analyzed using BD FACSDiva 8.

EGFR exon 20 cloning

Genomic DNA was extracted from PC9ER-AZDR cells using Pure Link genomic DNA Kit (Invitrogen). PCR reaction was performed using specific primers (forward: 5'-CACCATGCGAAGCCACACTG-3', and reverse: 5'-CTGGCTCCTTATCTCCCCTCC-3') and Iproof HF taq polymerase (BioRad). PCR products were cleaned and cloned into the pENTR/TOPO vector (Invitrogen) following the manufacture's instructions. After bacteria transformation and antibiotic selection, approximately 40 positive colonies were isolated. Plasmids were sequenced using M13 forward and M13 reverse primers.

Immunofluorescence

Cells were grown on sterile coverslips and fixed for 15 min at room temperature with phosphate-buffered saline (PBS) containing paraformaldehyde (4%). After rinsing, cells were permeabilized with Triton X-100 (0.5%) and blocked for 30 minutes at room temperature with bovine serum albumin (1%) and Tween 20 (0.2%). Coverslips were incubated overnight with γ-H2AX (1:200), p16 (1:200) or KI67 (1:200) antibodies in blocking buffer. The coverslips were rinsed in saline and then incubated with Alexa555-conjugated donkey anti-rabbit or anti-mouse secondary antibody. Cells were washed again in saline, counterstained with DAPI (Sigma-Aldrich) and mounted in ProLong mounting medium. Samples were examined using a Spinning Disk Confocal Microscope (Zeiss).

β-galactosidase staining

Detection of β-Gal activity was performed on frozen sections of tumor xenografts or on cultured cells. Sections were fixed in 0.5% glutaraldehyde (pH 7.4) in saline for 15 minutes, washed in saline supplemented with 1 mM $MgCl_2$ at pH 5.5, and stained for 5–6 hours at 37°C without CO_2 in saline containing 1mM $MgCl_2$ at pH 5.5, 1 mg/ml X-Gal, and 5 mM of each, potassium ferricyanide and potassium ferrocyanide. Thereafter, samples were washed twice and exposed for 30 minutes to formaldehyde (4%). Cryosections were additionally counterstained with Eosin. Photos were then taken using the Olympus SZX16 stereomicroscope.

Immunohistochemistry and histological staining

Mice were scarified and tumors were extracted and fixed in paraffin. Tissue sections were deparaffinized and rehydrated in an ethanol series. Antigen retrieval was performed at 100°C in citrate buffer (pH 6). Following blocking in 4% horse serum-containing solution (120 minutes), slides were incubated overnight with a primary antibody as follows: γ-H2AX (1:100), p21 (1:50), p16 (1:200) or KI67 (1:100). After incubation with HRP-conjugated secondary antibody (60 minutes at room temperature) staining was developed using the DAB kit (Vector Laboratories, Burlingame, CA), followed by Hematoxylin counterstaining (Sigma). Finally, slides were dehydrated and mounted with coverslips. Hematoxylin and Eosin (H&E) staining was performed on paraffin sections.

Immunofluorescence detection of receptor proteins in tumors

Tumors were harvested, formalin fixed and paraffin-embedded (FFPE). The corresponding sections (4 μm), obtained from each tissue block, were analyzed using immunofluorescence and an antibody to EGFR (Cell signaling, #4267S, 1:50), anti-HER-2 (Cell Signaling, #4290S, 1:50) or anti HER-3 (Cell Signaling #12708S, 1:50). FFPE tissues were deparaffinized in xylene and rehydrated in graded ethanol. Sections were incubated in acetone (-20 °C, 7 min) followed by antigen retrieval in Tris-EDTA solution (pH 9.0), in a microwave (for 10 minutes). After three washes in saline the slides were blocked in buffer containing 20% normal horse serum, followed by treatment with an avidin-biotin blocking solution for 15 minutes and then incubated overnight with the corresponding primary antibody. Slides were incubated for 24 hours at room temperature followed by incubation for 48 hours at 4ºC. Sections were washed and incubated with a biotinylated anti-rabbit secondary antibody for 90 minutes at room temperature, followed by a Cy3-conjugated streptavidin. Sections were washed with Aqua Polymount. Stained sections were examined and photographed using a fluorescence microscope (Eclipse Ni-U, Nikon, Tokyo, Japan) equipped with Plan Fluor objectives (20X), connected to a monochrome camera (DS-Qi1, Nikon).

Flow cytometry analyses of surface receptors

To determine surface receptor abundance, cells were treated with trypsin and washed twice in albumin containing saline (1% weight/volume). Thereafter, cells were incubated for 30 minutes at 4°C with anti-EGFR-FITC (1:200) , anti-HER2-APC (1:150) or anti-HER3-PE (1:100) antibodies. Cells were then washed twice and processed. The capability of the antibodies to bind with surface-localized receptors was correlated to the fluorescence intensity measured using the LSRII flow cytometer.
