# Supplementary material for: An oligoclonal antibody durably overcomes resistance of lung cancer to third‐generation EGFR inhibitors
Source: EMBO Mol Med. 2017 Dec 6;10(2):294–308. doi: 10.15252/emmm.201708076 (PMC5801506; doi:10.15252/emmm.201708076)
Supplement: Supplementary file 2 — Expanded View Figures PDF [file EMMM-10-294-s002.pdf]

Expanded View Figures

Figure EV1. A combination of three antibodies inhibits erlotinib-resistant lung cancer cells *in vitro* and in animals and downregulates both EGFR and phospho-EGFR.

A PC9ER (upper panel) and H1975 cells (lower panel) were grown in RPMI-1640 (2% serum) and exposed for 4 days to the indicated antibodies (20 μg/ml) against EGFR (cetuximab; CTX), HER2 (trastuzumab; TRZ), or HER3 (mAb33). Whenever antibody mixtures were applied, the total antibody concentration remained constant. Cell survival was assessed using the MTT colorimetric assay. Data are means ± SD. $^{**}P < 0.01$, and $^{***}P < 0.001$; $n = 3$; one-way ANOVA with Tukey's test.

B PC9 cells were cultured for 4 days with increasing concentrations of either TKIs (erlotinib, osimertinib, or CO-1686) or the triple antibody combination (CTX, TRZ, and mAb33). Metabolic activity was determined using the MTT assay. Data are means ± SD values from three experiments.

C PC9ER cells were treated for 24 h with saline, vehicle (DMSO), the indicated TKIs (each at 10 nM), mAb565 (20 μg/ml) against EGFR, cetuximab (CTX; 20 μg/ml), and two different antibody mixtures (3×mAbs): murine (M; mAbs 565, N12 and mAb33; each at 20 μg/ml) and partly human (H; CTX, TRZ and mAb33; each at 20 μg/ml). Thereafter, cells were analyzed using flow cytometry for surface expression levels of EGFR, HER2, and HER3. Normalized data are means ± SEM of two independent experiments.

D H1975 cells (3×10^6 cells per animal) were subcutaneously grafted in the flanks of CD1-nu/nu mice, which were subsequently randomized and subjected to the following treatments: erlotinib (50 mg/kg/day), osimertinib (5 mg/kg/day), or 3×mAbs (CTX, TRZ, and mAb33; 0.2 mg/mouse/injection; administered twice a week). Shown is immunohistochemical staining for KI67 in paraffin-embedded sections using specific antibodies. Scale bars, 100 μm.

E CD1-nu/nu mice harboring H1975 NSCLC xenografts were treated as indicated for 10 days. Thereafter, tumors were harvested, embedded in paraffin, and stained with antibodies specific to EGFR, EGFR-L858R, and phospho-EGFR (Y1068). Scale bars, 100 μm.

F Comparison of body weights (averages ± SD) of groups of eight CD1-nu/nu mice harboring H1975 xenografts and treated with either erlotinib (50 mg/kg/day), osimertinib (5 mg/kg/day), or a mixture of three mAbs (3×mAbs; CTX, TRZ, and mAb33; 0.2 mg/mouse/injection). Note that TKIs were daily administered using oral gavage, while the triple antibody combination was injected intraperitoneally once every 3 days.

G PC9ER cells (3×10^6 cells per animal) were subcutaneously grafted in the flanks of CD1-nu/nu mice. Animals were randomized into groups of six mice after tumors became palpable. Erlotinib (50 mg/kg/day) and osimertinib (5 mg/kg/day) were daily administered using oral gavage, while the triple antibody combination (3×mAbs; CTX, TRZ, and mAb33; 0.2 mg/mouse/injection) was administered intraperitoneally once every 3 days. Shown are results of body mass composition analyses (mean ± SD) of the fraction of fat mass (left) and lean mass (right) on day 20 of treatment. Mice harboring no tumors represent an internal control. One-way ANOVA with Tukey's test.

▶

 © 2017 The Authors

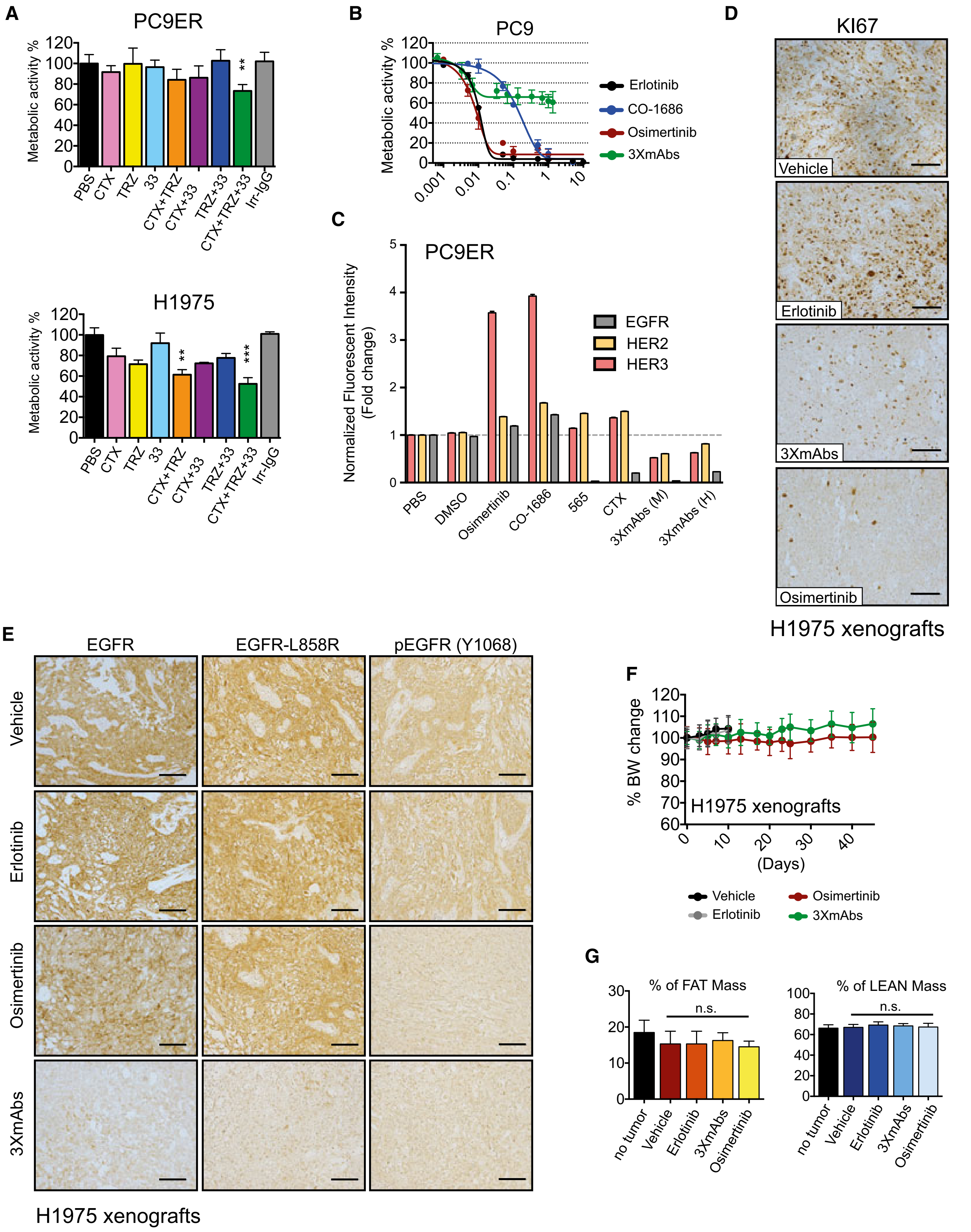
A
PC9ER
Metabolic activity %
PBS
CTX
TRZ
33
CTX+TRZ
CTX+33
TRZ+33
CTX+TRZ+33
Irr-IgG
H1975
B
PC9
Erlotinib
CO-1686
Osimertinib
3XmAbs
C
PC9ER
Normalized Fluorescent Intensity (Fold change)
EGFR
HER2
HER3
DMSO
565
3XmAbs (M)
3XmAbs (H)
D
KI67
Vehicle
H1975 xenografts
E
EGFR
EGFR-L858R
pEGFR (Y1068)
F
% BW change
(Days)
G
% of FAT Mass
% of LEAN Mass
n.s.
no tumor

Figure EV1.

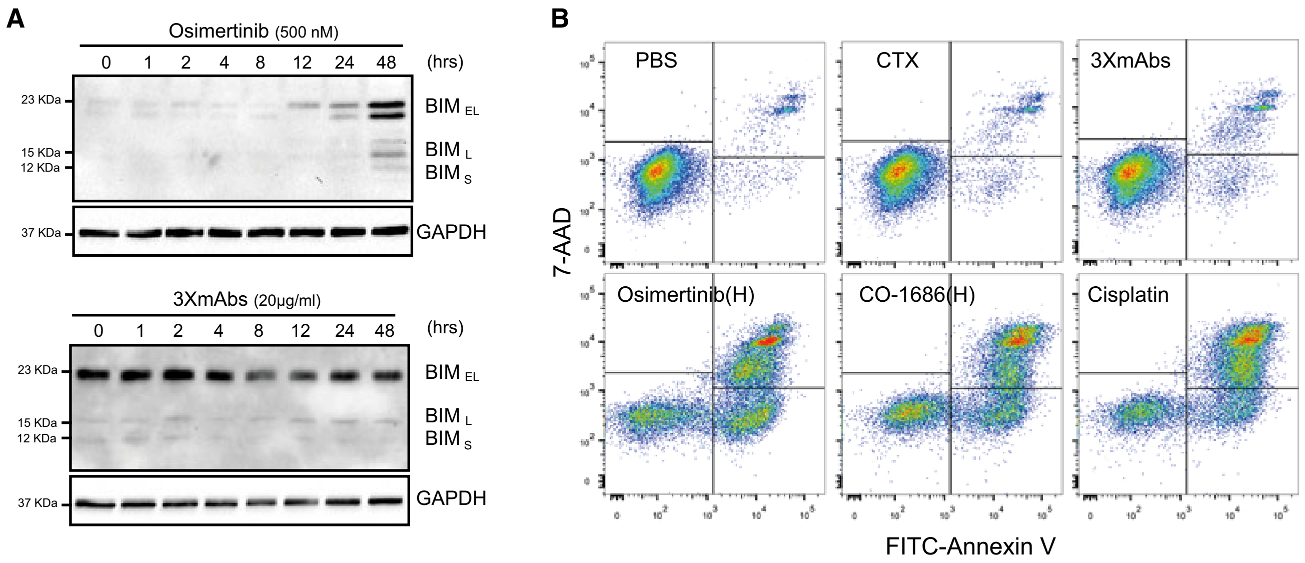

Figure EV2. Unlike antibodies, kinase inhibitors induce apoptosis of mutant EGFR expressing cells.

A PC9-ER cells were treated with osimertinib (500 nM; upper panel) or with 3×mAbs (20 μg/ml; lower panel) for the indicated time intervals. Cleared cell extracts were subjected to immunoblotting with an anti-BIM antibody. GAPDH was used as a loading control. The three forms of BIM are indicated.

B PC9ER cells were treated for 48 h with the following agents: saline (PBS), cetuximab (CTX, 20 μg/ml), 3×mAbs (CTX, TRZ, and mAb33, each at 20 μg/ml), osimertinib (0.5 μM), CO-1686 (0.5 μM), and cisplatin (1 μM). Shown are results of an apoptosis assay performed using an annexin V/7-AAD kit (BioLegend, Inc). Quantification of the fractions of early and late apoptotic cells are shown in Fig 2C. The experiment was repeated three times.

Source data are available online for this figure.

Figure EV3. Treatment with a triple antibody combination associates with phenotypic attributes of cellular senescence.

A Beta-galactosidase (β-Gal) staining of H1975 cells pre-treated for 11 days with either saline or 3×mAbs (CTX, TRZ, and mAb33; total = 20 μg/ml). Scale bar, 100 μm.

B Whole extracts were prepared from H1975 cells that were pre-exposed to 3×mAbs for the indicated number of days. Following gel electrophoresis and transfer to filters, we probed for the indicated antigens, including KIP/p27, a cell cycle inhibitor.

C, D Immunohistochemical staining of paraffin-embedded sections from H1975 xenografts. One week after tumor inoculation, mice were randomized (3–4 mice/group) and treated for 14 days with vehicle, 3×mAbs (CTX, TRZ, and mAb33; 0.2 mg/mouse/injection, once every 3 days), or osimertinib (5 mg/kg/dose; once daily). Sections were stained with p21- or γH2AX-specific antibodies. Scale bars, 100 μm. The bar plots depict quantifications of antigen staining (mean ± SD). *$P < 0.05$; ***$P < 0.001$; $n = 3$; one-way ANOVA with Tukey's comparison.

E Shown are immunoblots of PC9ER cells exposed for 4 days to treatments with the indicated antibodies and their combinations. Total antibody concentrations were kept identical (20 μg/ml). Tubulin served as a loading control. The following treatments were used: PBS, saline solution; 565, a murine antibody against EGFR; 12, a murine antibody against HER2; 33, a murine antibody against HER3; CTX, cetuximab; TRZ, trastuzumab; Irr-IgG: irrelevant immunoglobulin G.

F PC9ER or H1975 cells were treated for 9 days with either saline (PBS; blue bars) or 3×mAbs (gray bars). qPCR analysis was performed using primers corresponding to the indicated transcripts. Shown are averages ± SD. *$P < 0.05$; $n = 3$; *t*-test.

G Mice were grafted with H1975 cells and randomized into groups of three mice, which were treated as follows: vehicle, erlotinib (50 mg/kg/day), osimertinib (5 mg/kg/day), or 3×mAbs (CTX, TRZ, and mAb33; 0.2 mg/mouse/injection). Two weeks later, tumors were stained with hematoxylin and eosin. The squares in the left-hand photographs are magnified and shown in the right-hand panels. Arrowheads indicate tumor infiltrating cells. Scale bars, 100 μm.

H H1975 cells were treated with saline, osimertinib, CTX (20 μg/ml), or 3×mAbs (total: 20 μg/ml) before adding natural killer (NK) cells. Alternatively, human NK cells were added 9 days later (panel labeled: pretreatment), and cell indices were determined using a real-time cell analyzer (RTCA). Data are means ± SD values. *$P < 0.05$; **$P < 0.01$; ***$P < 0.001$; $n = 3$; two-way ANOVA with Bonferroni's comparison test. Tx-100 refers to Triton X-100.

I CD1-nu/nu mice were inoculated with H1975 lung cancer cells (4×10^6 cells). Once tumors reached approximately 500 mm^3, groups of 3–4 mice were treated for 2 weeks with vehicle, 3×mAbs (CTX, TRZ, and mAb33; 0.2 mg/mouse/injection, once every 3 days), or with osimertinib (5 mg/kg/day). Tumors were snap-frozen, and cryosections were probed for beta-galactosidase (β-Gal) and counterstained using nuclear fast red. The inset shows an enlarged area. Scale bar, 200 μm.

Source data are available online for this figure.

 © 2017 The Authors

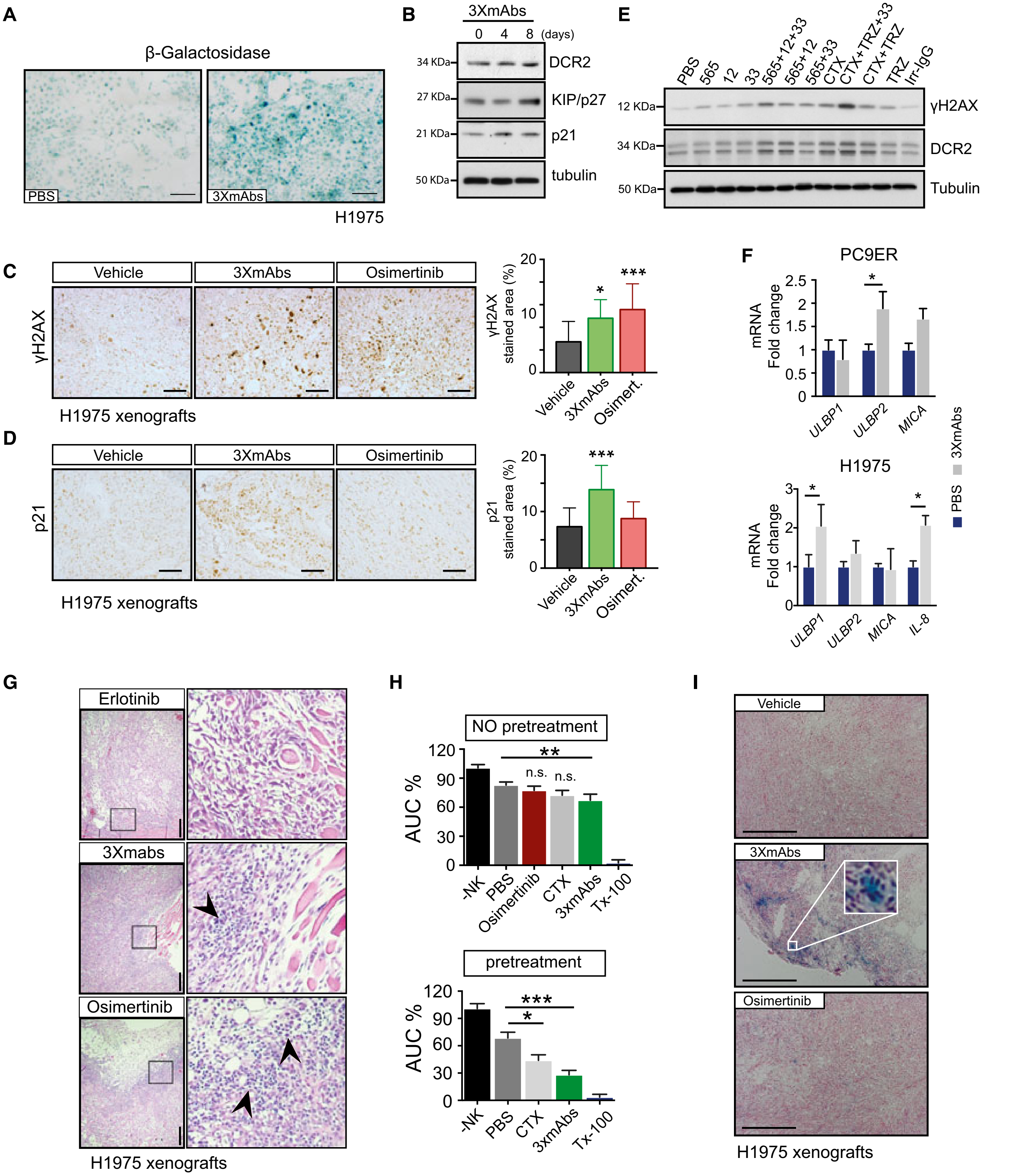
A
β-Galactosidase
PBS
3XmAbs
H1975
B
3XmAbs
0 4 8 (days)
34 KDa
DCR2
27 KDa
KIP/p27
21 KDa
p21
50 KDa
tubulin
E
PBS 565 12 33 565+12+33 565+12 565+33 CTX CTX+TRZ+33 CTX+TRZ TRZ Irr-IgG
12 KDa
γH2AX
34 KDa
DCR2
50 KDa
Tubulin
C
Vehicle
3XmAbs
Osimertinib
γH2AX
H1975 xenografts
γH2AX stained area (%)
Vehicle
3XmAbs
Osimert.
D
p21
p21 stained area (%)
F
PC9ER
mRNA Fold change
ULBP1
ULBP2
MICA
H1975
IL-8
3XmAbs
PBS
G
Erlotinib
3Xmabs
Osimertinib
H
NO pretreatment
AUC %
n.s.
-NK
PBS
Osimertinib
CTX
3xmAbs
Tx-100
pretreatment
I
Vehicle
3XmAbs
Osimertinib

Figure EV3.

Figure EV4. Cooperative anti-tumor effects of the combination of osimertinib and 3×mAbs.

A Comparison of body weights (averages ± SD) of groups of eight CD1-nu/nu mice harboring PC9ER xenografts, which were treated with either erlotinib (50 mg/kg/day), osimertinib (High; 5 mg/kg/day), or a mixture of three mAbs (3×mAbs; CTX, TRZ, and mAb33; 0.2 mg/mouse/injection). Note that TKIs were daily administered using oral gavage, whereas the triple antibody combination was injected intraperitoneally once every 3 days.

B Shown are results of body mass composition analyses (mean ± SD) of the fraction of fat mass (left) and lean mass (right) on day 20 of treatment. Mice harboring no tumors were used as an internal control.

C Nude mice harboring PC9ER tumor xenografts were treated with osimertinib and 3×mAbs. The dot plot presents tumor volumes determined 83 days after the beginning of treatments. Note that each symbol represents a single animal. Data are means (± SD) from 9 to 10 mice per group. The experiment was performed once and each arm included 8–10 mice. The horizontal bars indicate medians and the upper and lower quartiles.

D PC9ER cells were treated for 24 h with the indicated concentrations of osimertinib, either alone or in combination with 3×mAbs (CTX, TRZ, and mAb33; 30 μg/ml). Cell extracts were prepared, electrophoresed, and immunoblotted for EGFR, phospho-EGFR (Y1068), caspase-3 and cleaved caspase-3, PARP, and its cleaved forms. Tubulin was used as an equal loading control. Blots are representative of two experiments. The cleaved forms (cl.) of PARP and caspase-3 are indicated, along with the corresponding molecular weight markers.

E PC9ER cells were pre-treated for 11 days with 3×mAb (30 μg/ml). Thereafter, both pre-treated cells and control cells were incubated for 24 h with 3×mAbs (20 μg/ml), either in the absence or in the presence of the indicated concentrations of osimertinib. Whole-cell extracts were probed for caspase-3 and its cleaved form (cleaved caspase-3). "Long exp." refers to a longer film exposure aimed at enhancing the signals. Tubulin served as an equal loading control.

F PC9ER cells were treated with osimertinib (500 nM), 3×mAbs (20 μg/ml), or with the respective combination for the indicated time intervals. Cell extracts were subjected to immunoblotting with anti-BIM antibodies. GAPDH was used as a loading control. The three forms of BIM are indicated, along with the corresponding molecular weight markers.

G PC9ER cells were treated for 24 h with 3×mAbs (30 μg/ml), osimertinib (500 nM), or a combination of the two treatments. DMSO was used as a control treatment (CTRL). Following incubation with BrdU (60 min), cells were fixed and subjected to BrdU and PI staining. Shown are cell cycle distributions of a representative experiment performed on 200,000 cells/sample using cytometry. The experiment was repeated twice.

Source data are available online for this figure.

© 2017 The Authors

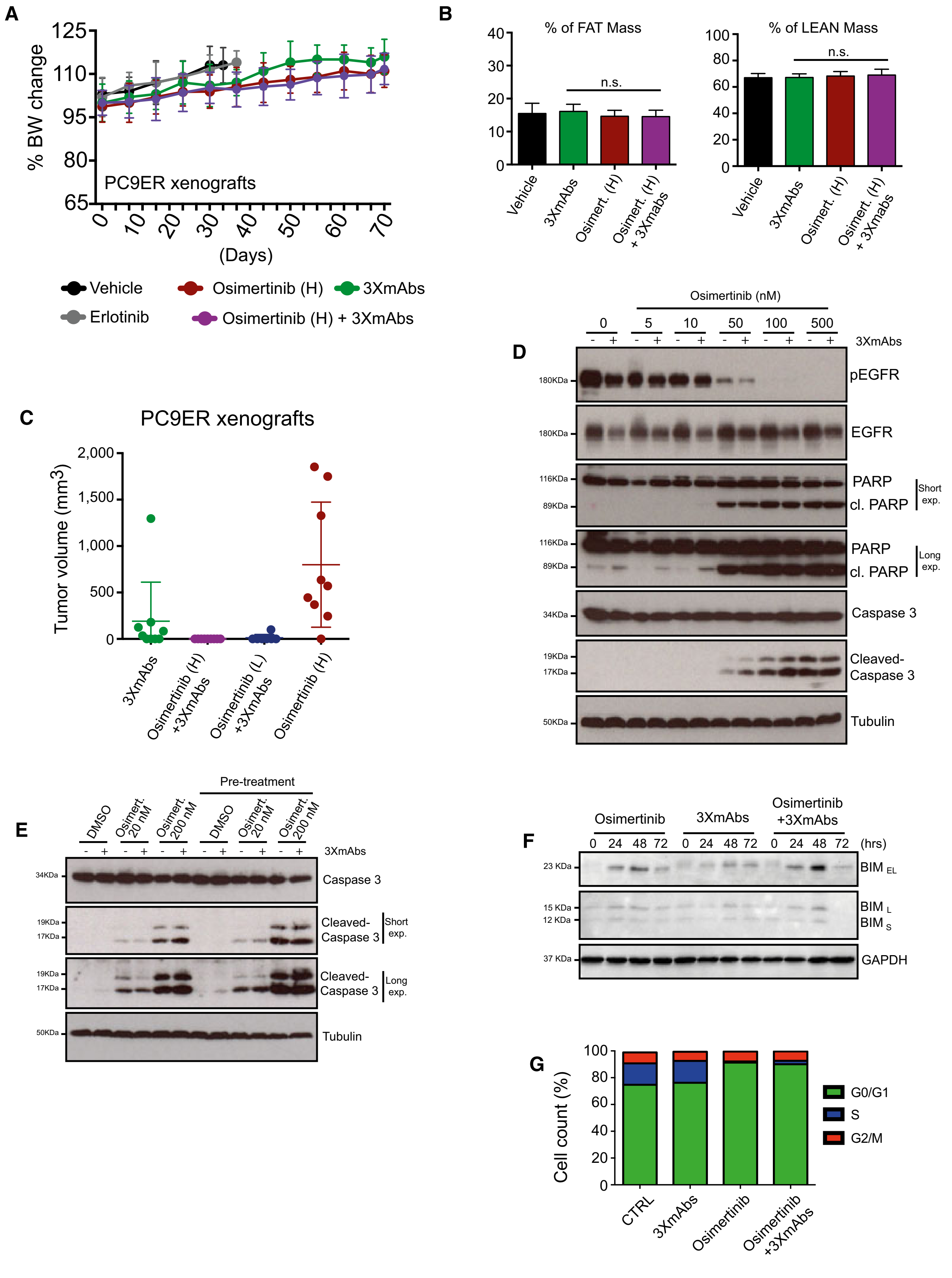
A
% BW change
PC9ER xenografts
(Days)
Vehicle
Erlotinib
Osimertinib (H)
Osimertinib (H) + 3XmAbs
3XmAbs
B
% of FAT Mass
% of LEAN Mass
n.s.
Vehicle
3XmAbs
Osimert. (H)
Osimert. (H) + 3Xmabs
C
PC9ER xenografts
Tumor volume (mm3)
3XmAbs
Osimertinib (H) +3XmAbs
Osimertinib (L) +3XmAbs
Osimertinib (H)
D
Osimertinib (nM)
3XmAbs
pEGFR
EGFR
PARP
cl. PARP
Short exp.
Long exp.
Caspase 3
Cleaved-Caspase 3
Tubulin
E
Pre-treatment
DMSO
Osimert. 20 nM
Osimert. 200 nM
F
Osimertinib
3XmAbs
Osimertinib +3XmAbs
(hrs)
BIM EL
BIM L
BIM S
GAPDH
G
Cell count (%)
CTRL
G0/G1
S
G2/M

Figure EV4.

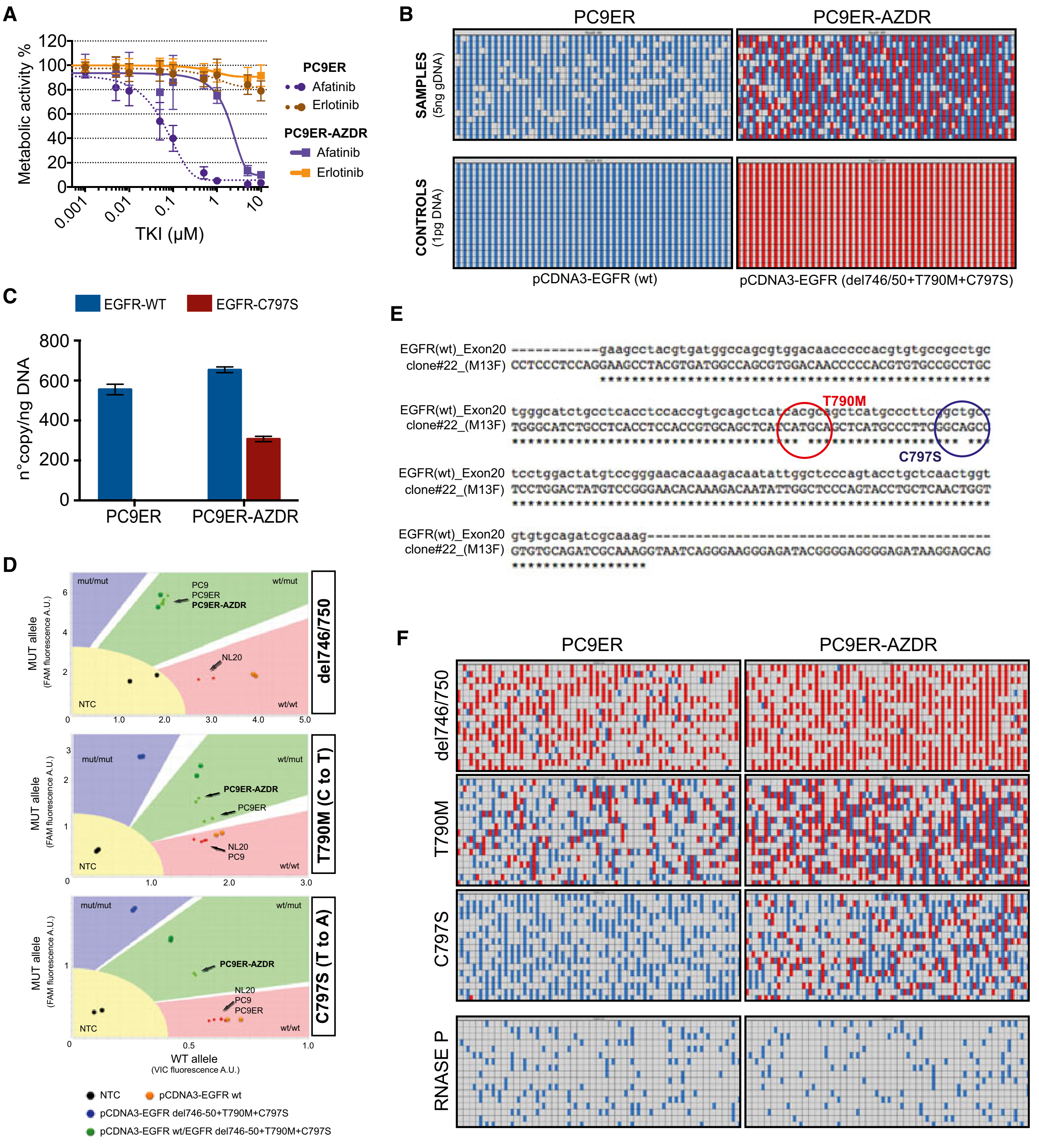

Figure EV5.

 © 2017 The Authors

Figure EV5. *In vitro* acquired resistance to osimertinib associates with emergence of the C797S mutation and resistance to afatinib.

A Shown are survival assays of PC9ER and derivative PC9ER-AZDR cells, which were selected *in vitro* for resistance to osimertinib. Cells were treated for 72 h with increasing concentrations of erlotinib or afatinib, and MTT signals were determined. Data are means ± SD values of three independent experiments.

B Genomic DNA was isolated from PC9ER and from PC9ER-AZDR cells and then subjected to dPCR analysis using the Fluidigm Biomark platform. Thereafter, DNA stocks were diluted to 10 ng/µl. In parallel, DNA was isolated from control pcDNA3 plasmids, encoding either wild-type EGFR or the triple mutant del746/50, T790M, and C797S. Five serial dilutions of the two genomic DNA samples were prepared and loaded onto the dPCR chip, side by side with DNA from plasmids (1 pg). The results obtained with one serial dilution (5 ng DNA) and C797S-specific primers are presented alongside the results from control plasmids. Analysis was performed utilizing dPCR software from Fluidigm. Amplifications with mutation positive and mutation negative wells appear in red and in blue, respectively.

C Histograms depicting results of digital PCR (dPCR) analysis performed three times with genomic DNA isolated from PC9ER and PC9ER-AZDR cells. Average numbers of copies per 1 ng of genomic DNA (± SD) were obtained from serial dilutions of DNA.

D The TaqMan genotyping assay was applied on genomic DNA isolated from PC9, PC9ER, and PC9ER-AZDR cells. As reference, we used DNA from normal human lung epithelial cells (NL20), along with a plasmid DNA encoding EGFR harboring the indicated mutations. Fluorescent signals corresponding to cellular DNA samples are shown as small colored dots, whereas the larger dots represent signals obtained from plasmid DNAs. Note that VIC-labeled primers were used for amplification of wild-type alleles, whereas FAM-labeled primers were used for the mutant alleles. Each sample was run in duplicates. Data were analyzed using TaqMan Genotyper Software (Life technologies). As indicated, PC9ER-AZDR cells scored positive for three mutant alleles: del746/750, T790M, and C797S. NTC, no-template control.

E Genomic DNA was extracted from PC9ER-AZDR cells. The region corresponding to EGFR's exon 20 was sequenced and aligned with the WT sequence. Note that the C to T (T790M) mutation (red) and the T to A (C797S) mutation (blue) are allelic.

F Genomic DNA isolated from PC9ER and from PC9ER-AZDR cells was subjected to dPCR analysis using the Fluidigm Biomark platform (gDNA, 2.5 ng/sample). Exact copy number of the indicated EGFR mutated alleles, as well as the WT alleles, was then assessed. The RNase P specific set of probes was used as normalizer. The analysis was performed utilizing the dPCR software from Fluidigm. Amplifications with mutation positive and mutation negative wells appear in red and in blue, respectively.
