# Supplementary material for: An oligoclonal antibody durably overcomes resistance of lung cancer to third‐generation EGFR inhibitors
Source: EMBO Mol Med. 2017 Dec 6;10(2):294–308. doi: 10.15252/emmm.201708076 (PMC5801506; doi:10.15252/emmm.201708076)
Supplement: Supplementary file 3 — Source Data for Expanded View [file EMMM-10-294-s003.zip › EMM_8076_Source_data_Fig_EV3.pdf]

Figure EV3b

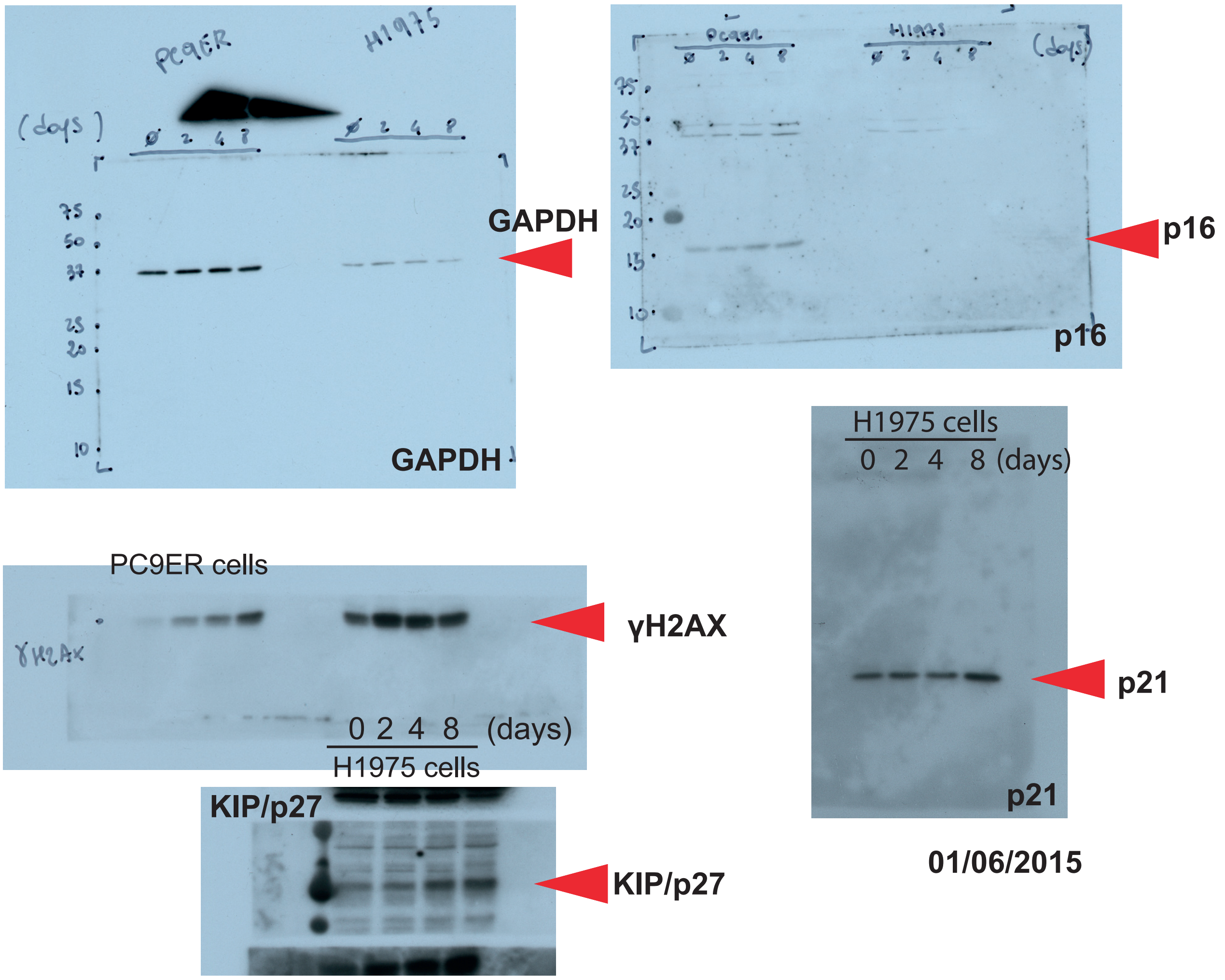

Blots represent a second repeatition of the experiment shown in Fig. EV3B

Figure EV3e

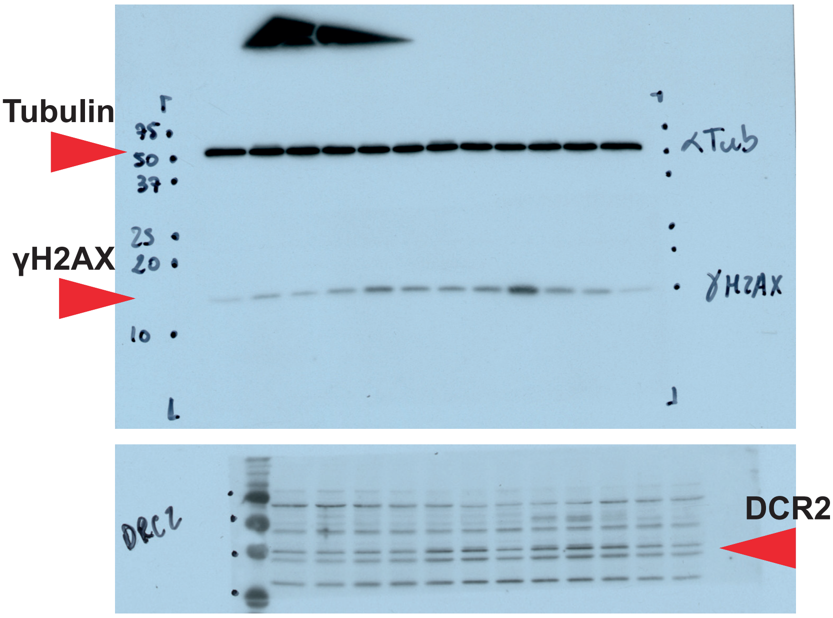

14/06/2015

Figure EV3g

Erlotinib 3xmAbs Osimertinib

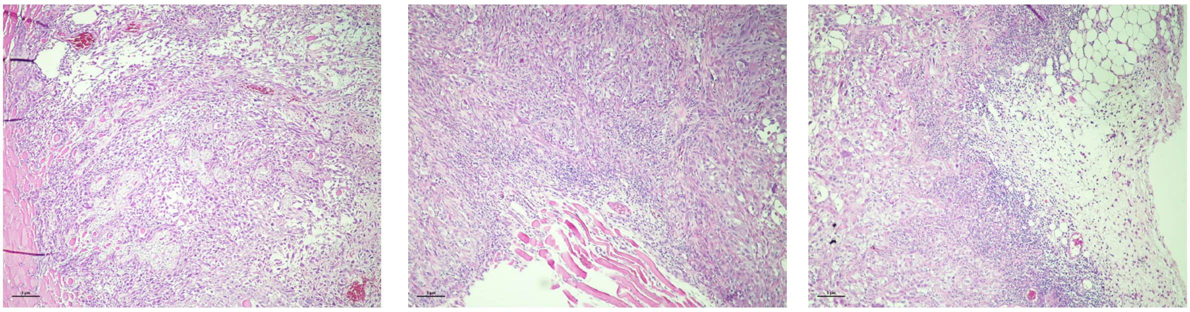

10/09/2015
