# Supplementary material for: An oligoclonal antibody durably overcomes resistance of lung cancer to third‐generation EGFR inhibitors
Source: EMBO Mol Med. 2017 Dec 6;10(2):294–308. doi: 10.15252/emmm.201708076 (PMC5801506; doi:10.15252/emmm.201708076)
Supplement: Supplementary file 3 — Source Data for Expanded View [file EMMM-10-294-s003.zip › EMM_8076_Source_Data_Fig_EV4.pdf]

Figure EV4d

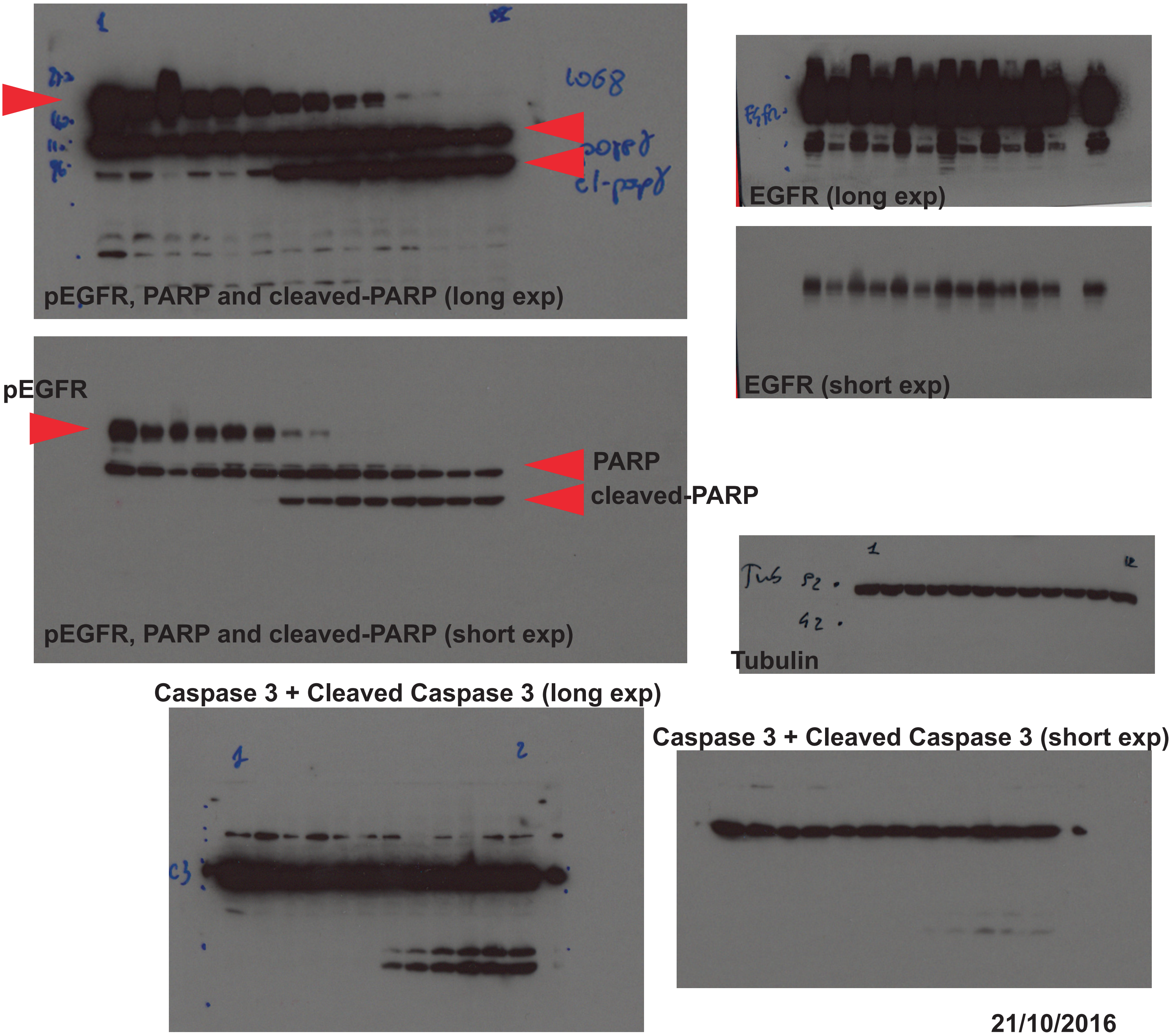

21/10/2016

Figure EV4e

Casp. 3

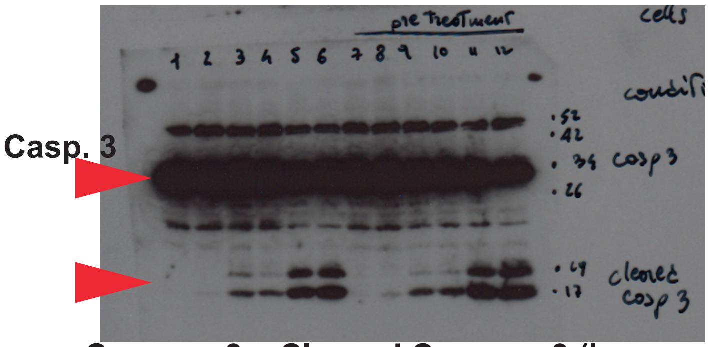

Caspase 3 + Cleaved Caspase 3 (long exp)

Casp. 3

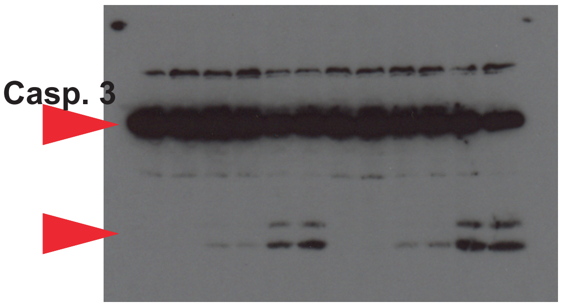

Caspase 3 + Cleaved Caspase 3
(medium exp)

Casp. 3

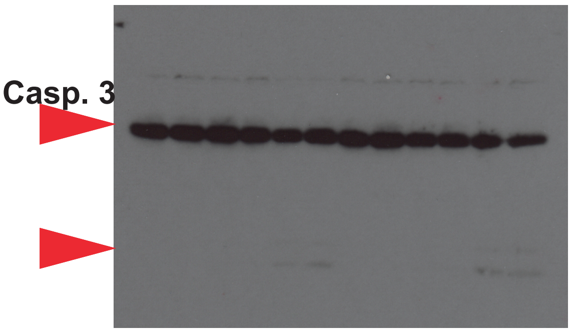

Caspase 3 + Cleaved Caspase 3
(short exp)

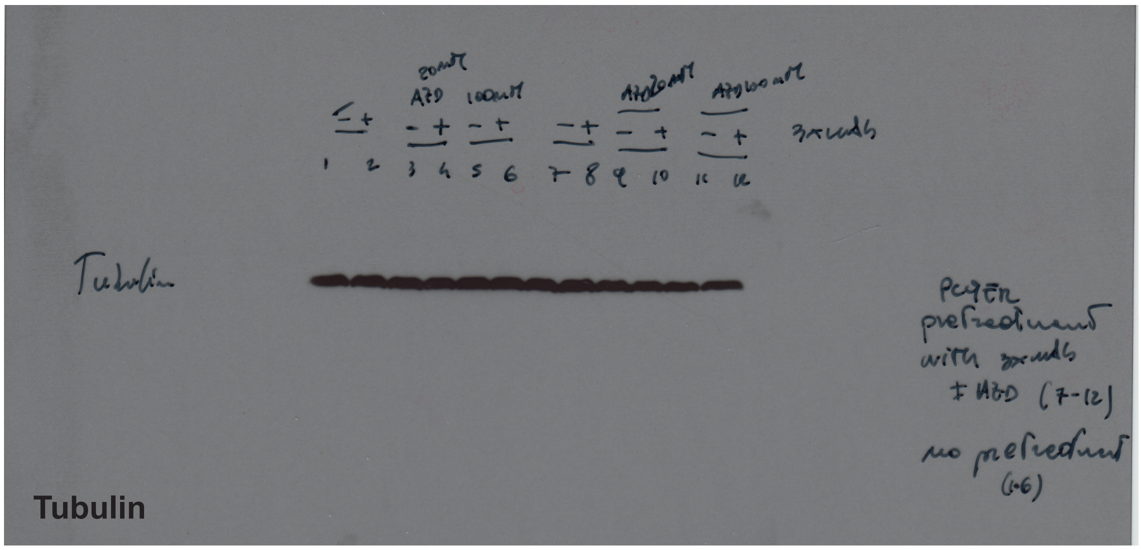

Tubulin

22/11/2016

Figure EV4f

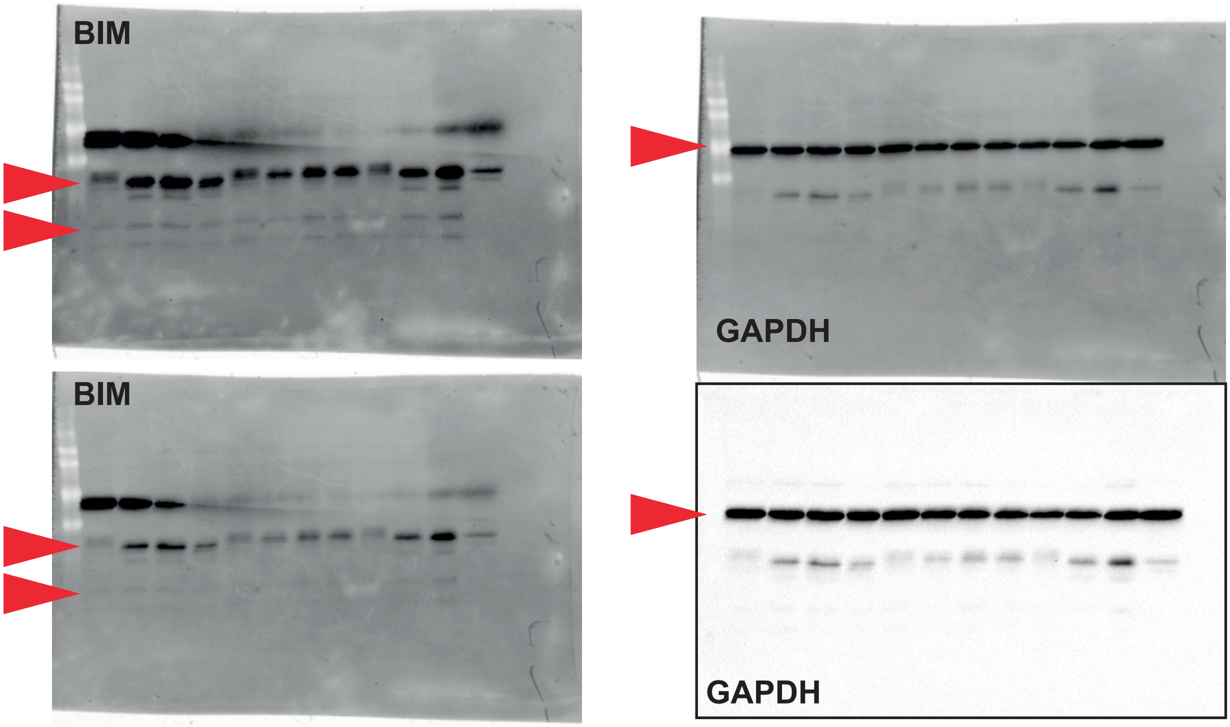

02/12/2016
