# Supplementary figures and images for: An oligoclonal antibody durably overcomes resistance of lung cancer to third‐generation EGFR inhibitors
Source: EMBO Mol Med. 2017 Dec 6;10(2):294–308. doi: 10.15252/emmm.201708076 (PMC5801506; doi:10.15252/emmm.201708076)

Figure EV2a

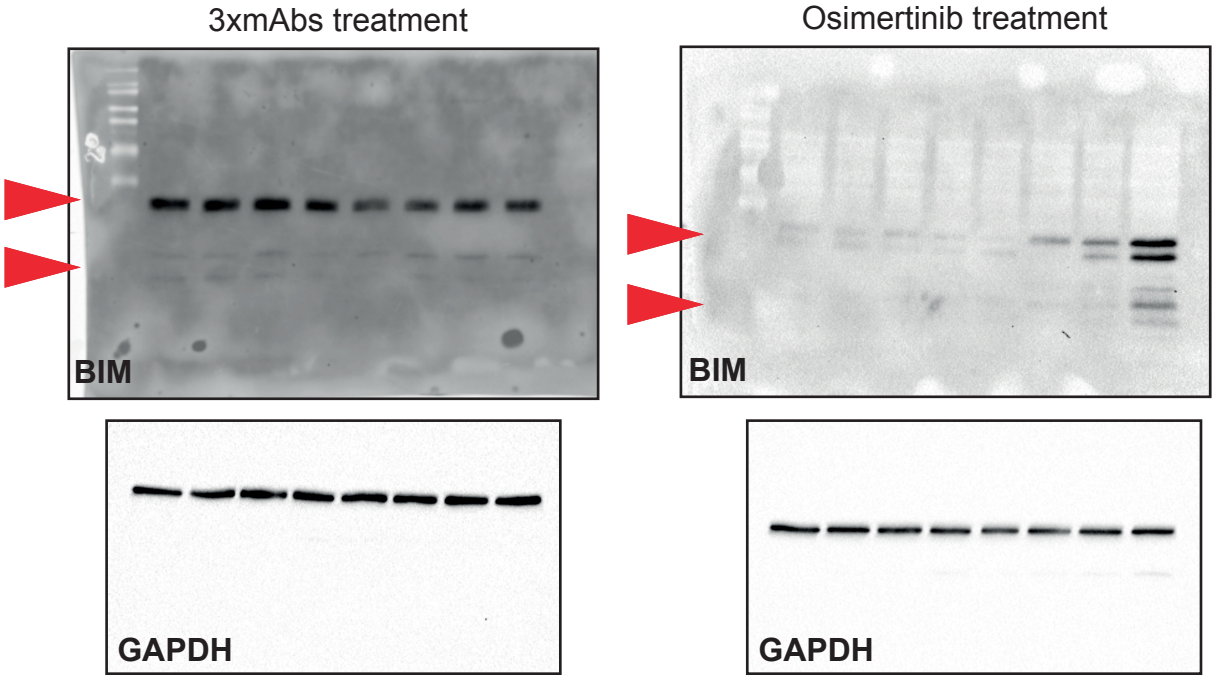

02/12/2016

Supplement: Supplementary file 3 — Source Data for Expanded View [file EMMM-10-294-s003.zip › EMM_8076_Source_Data_Fig_EV2.pdf]

Figure 2B

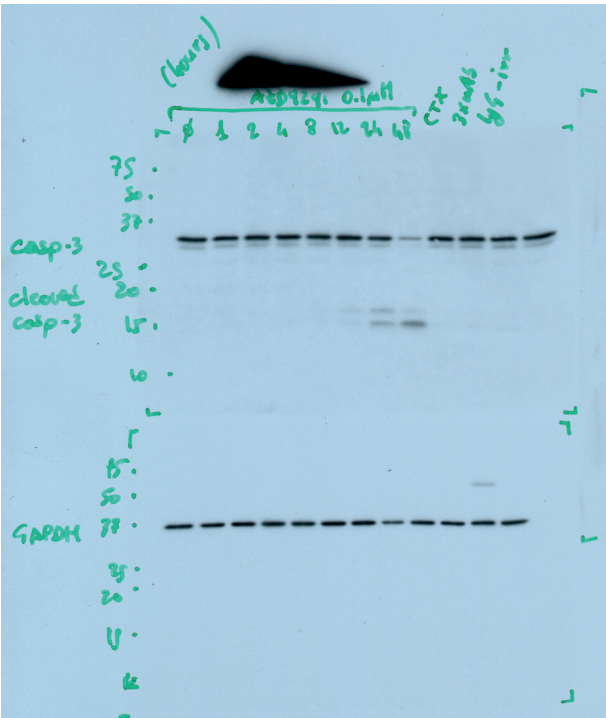

19/05/2015

Caspase 3 and Cleaved-caspase 3

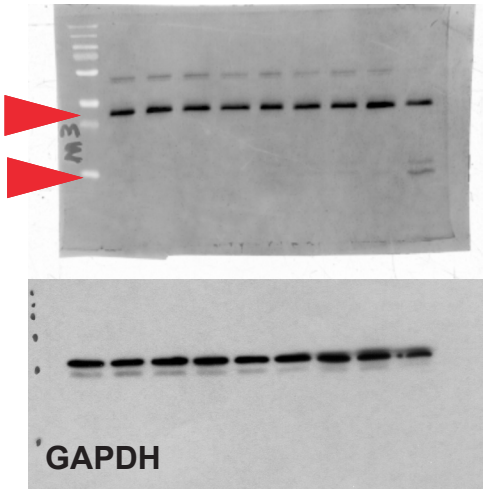

11/07/2017

Figure 2D

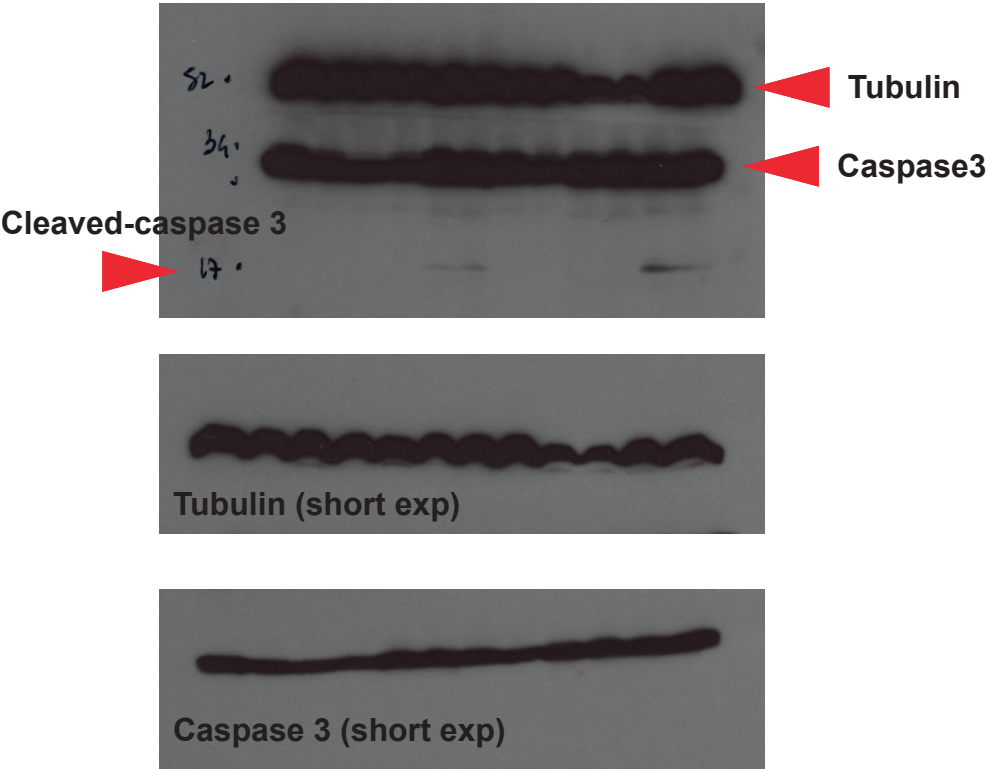

22/11/2016

Supplement: Supplementary file 6 — Source Data for Figure 2 [file EMMM-10-294-s005.pdf]

**Figure 3B**

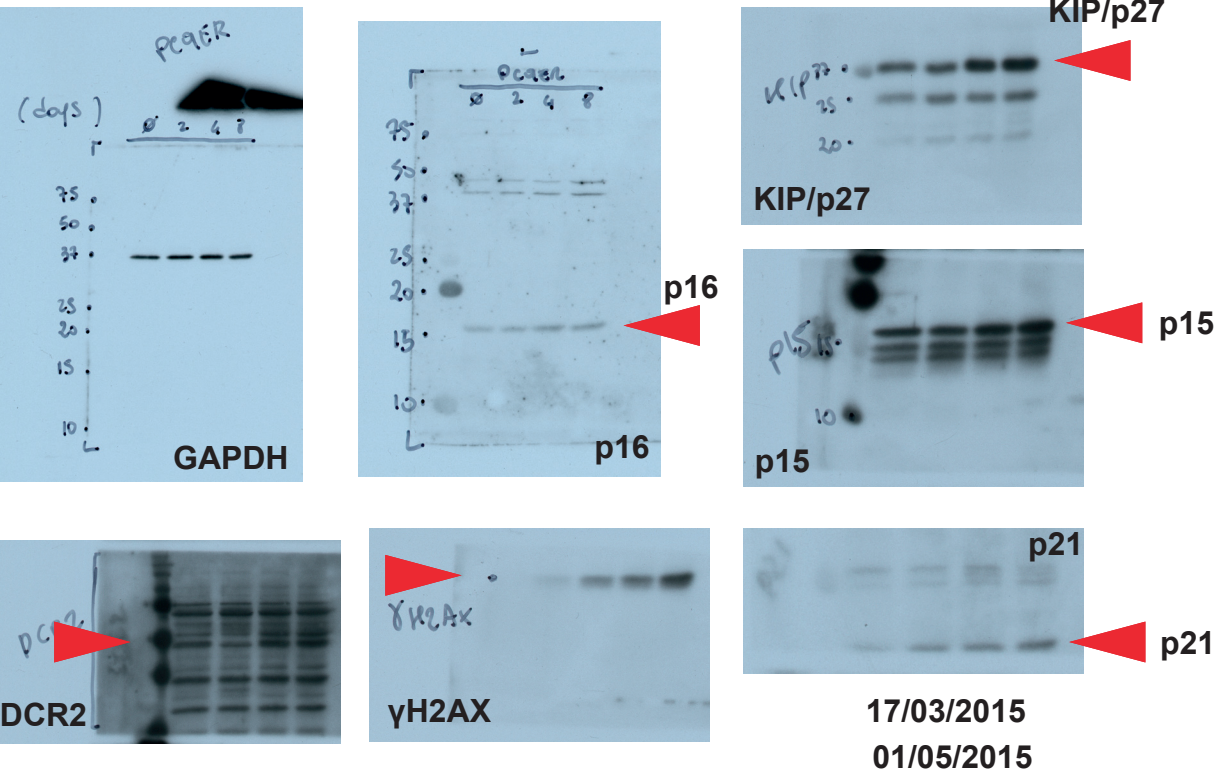

Supplement: Supplementary file 7 — Source Data for Figure 3 [file EMMM-10-294-s006.pdf]

Figure 5A

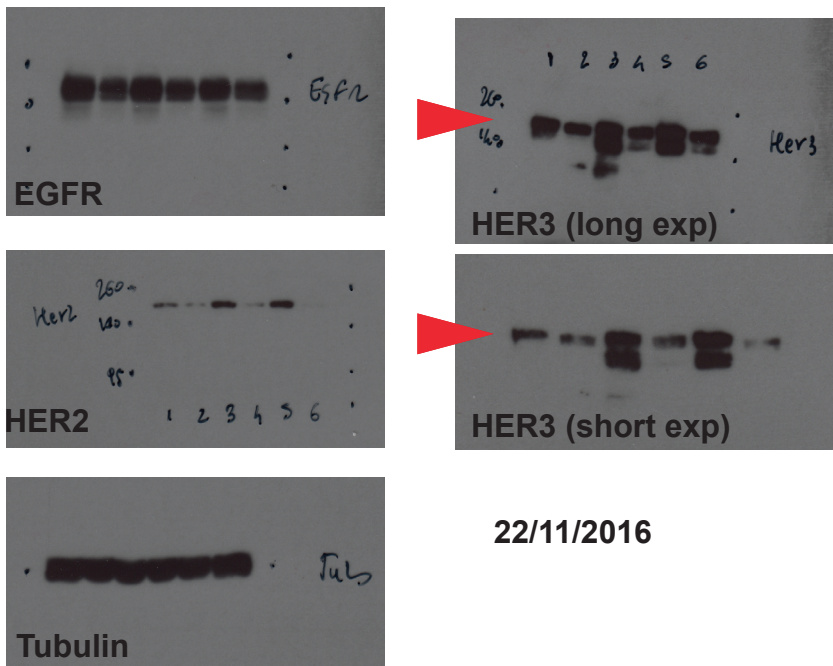

Figure 5C

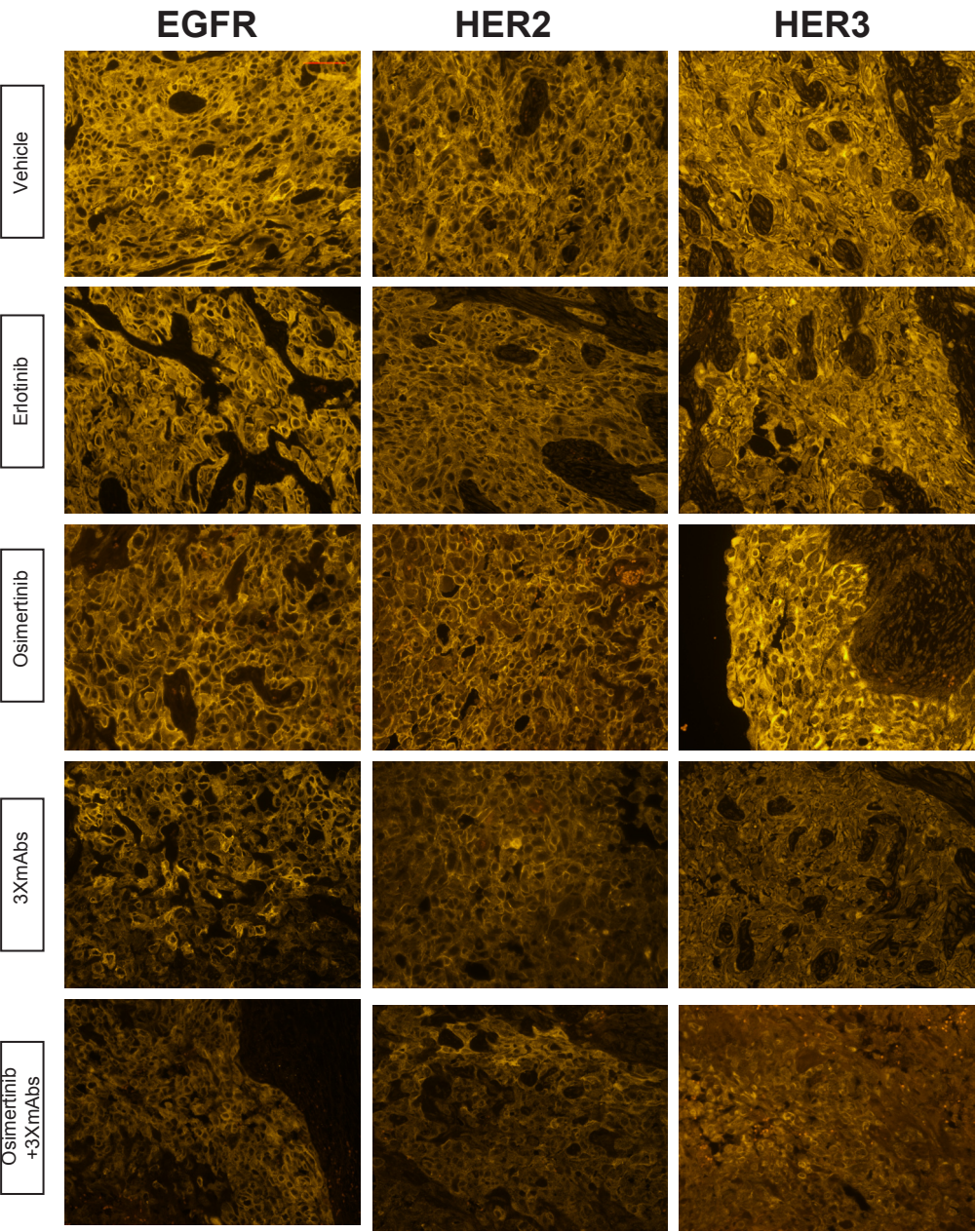

**Figure 5D**

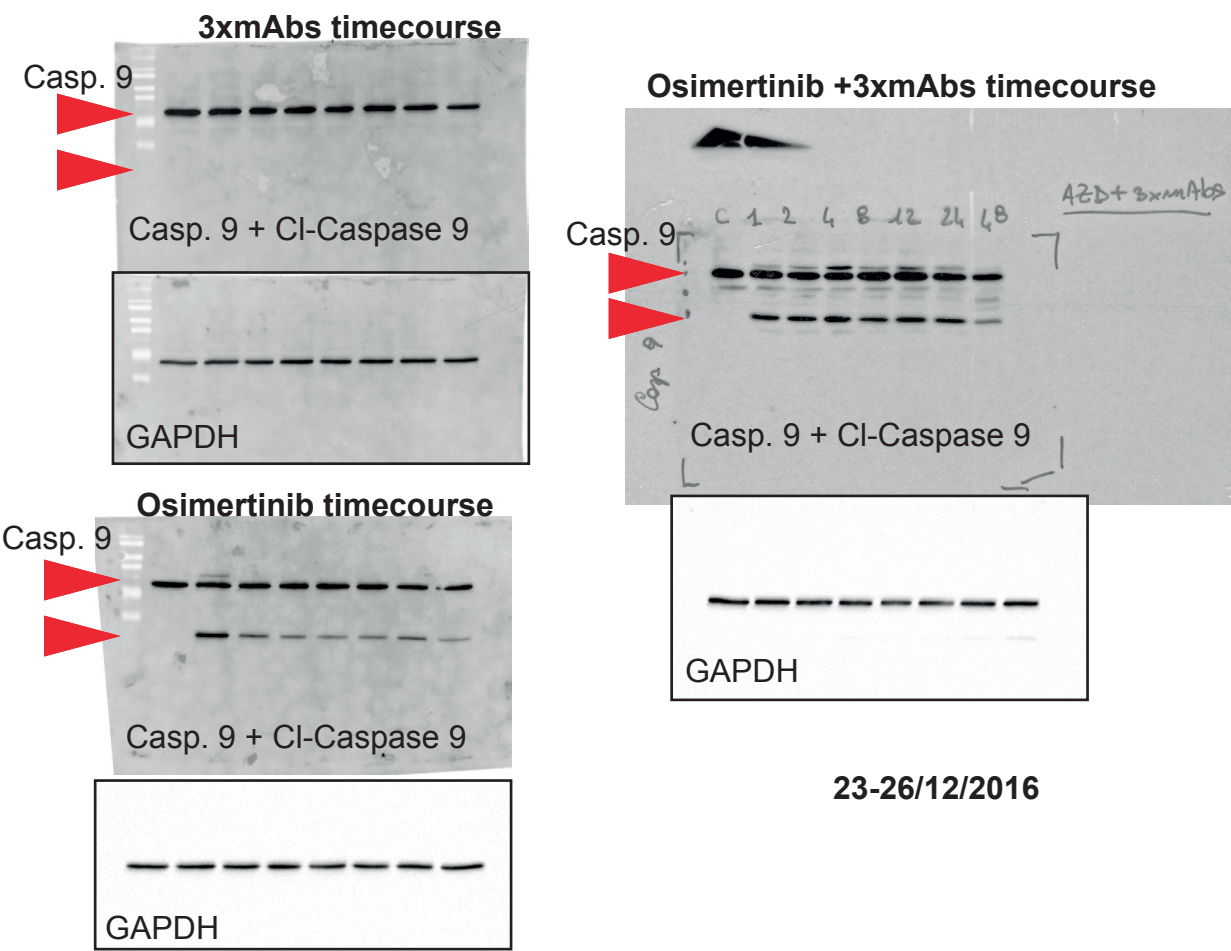

**Figure 5E**

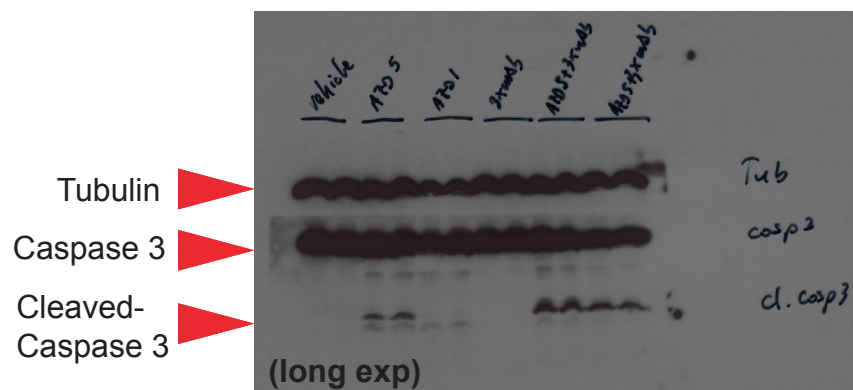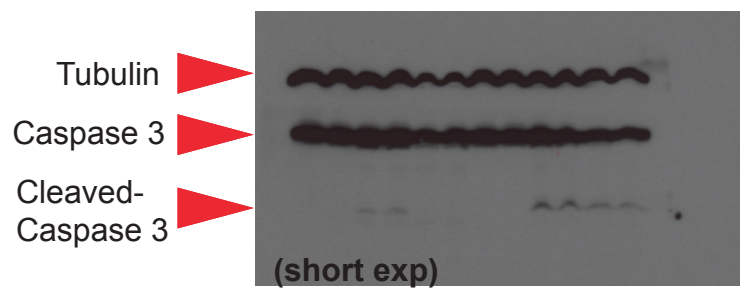

23/11/2016

Supplement: Supplementary file 8 — Source Data for Figure 5 [file EMMM-10-294-s007.pdf]

Figure 6B

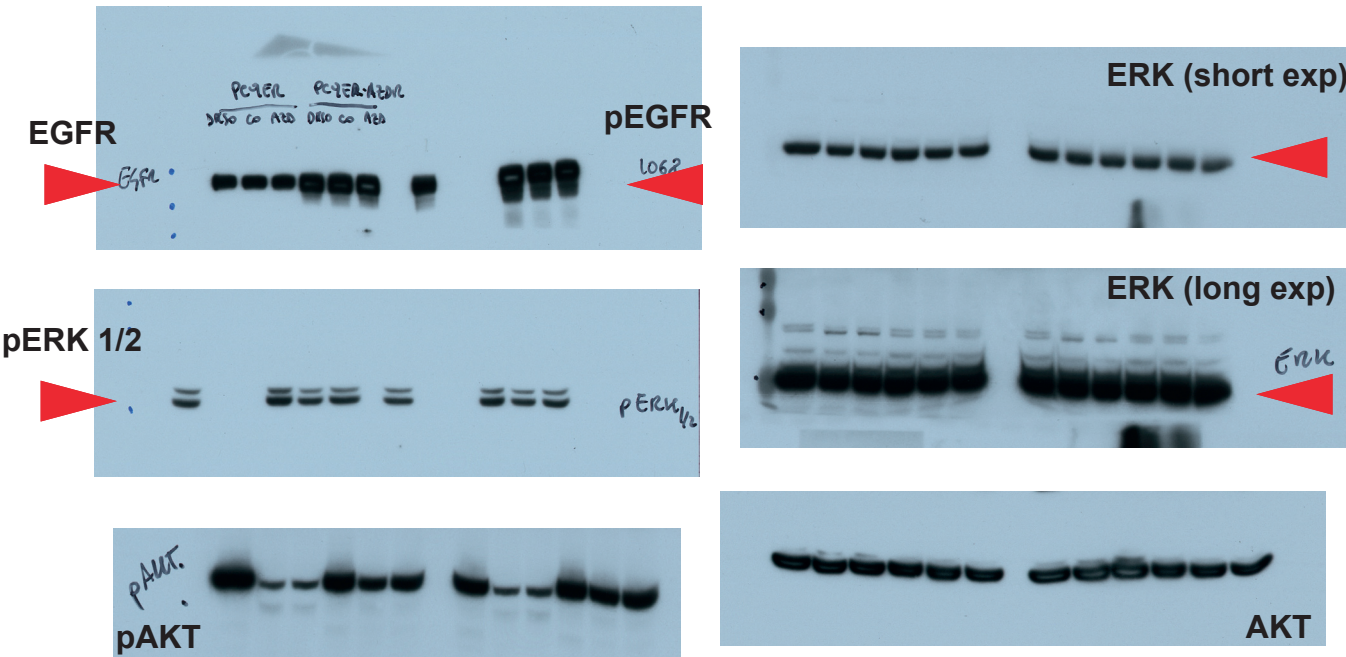

03/07/2015

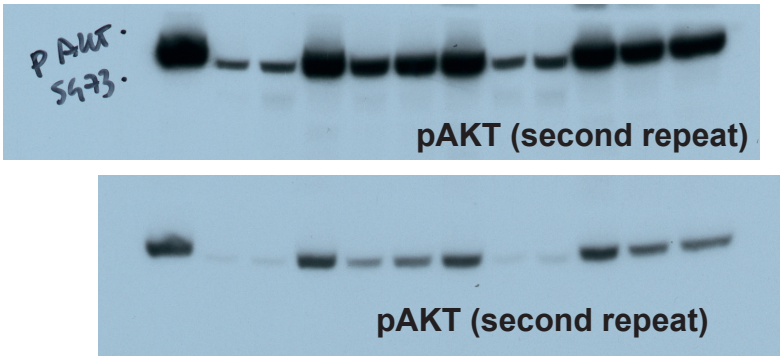

(second repeat) 06/07/2015

Supplement: Supplementary file 9 — Source Data for Figure 6 [file EMMM-10-294-s008.pdf]
