# Supplementary material for: An oligoclonal antibody durably overcomes resistance of lung cancer to third‐generation EGFR inhibitors
Source: EMBO Mol Med. 2017 Dec 6;10(2):294–308. doi: 10.15252/emmm.201708076 (PMC5801506; doi:10.15252/emmm.201708076)
Supplement: Supplementary file 5 — Source Data for Figure 1 [file EMMM-10-294-s004.pdf]

Figure 1C

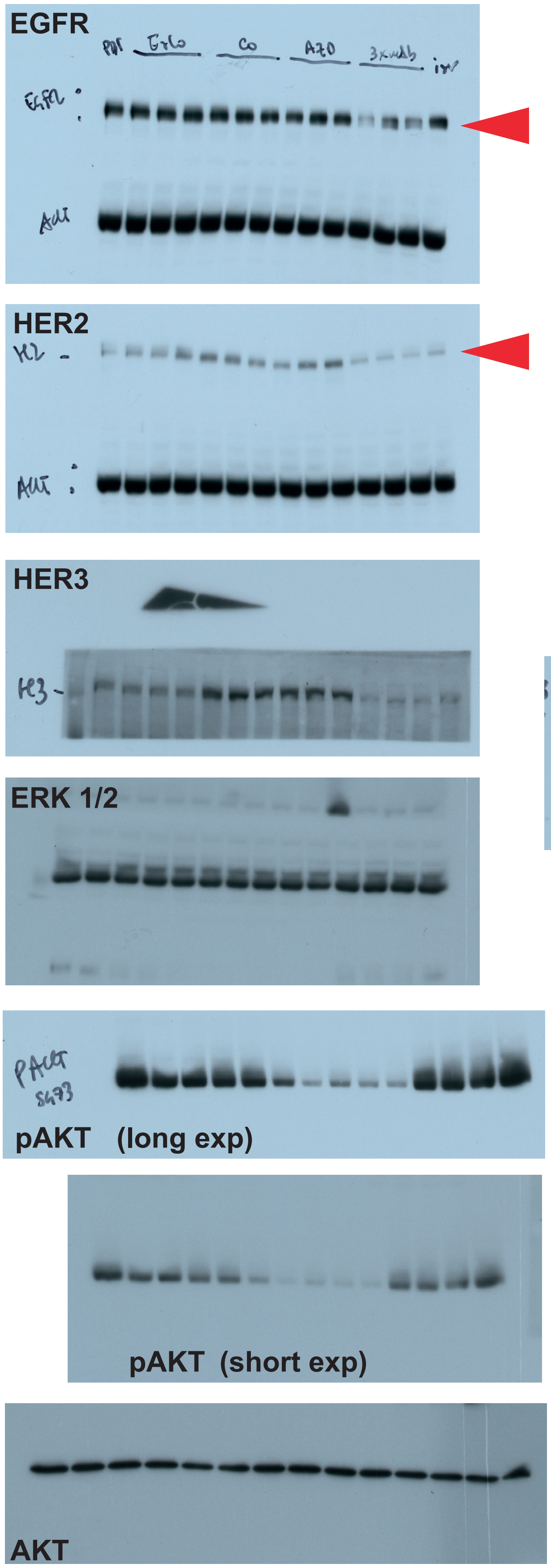

01/06/2015

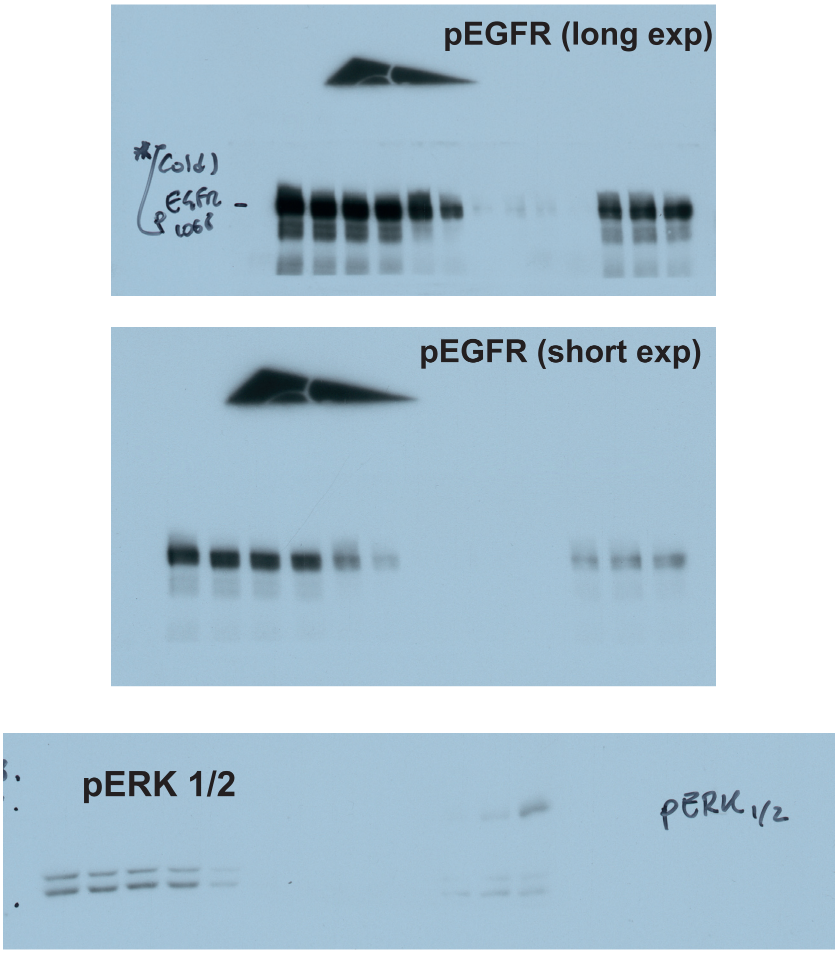

03/06/2015

***Please note:**

the “Irrelevant antibody” conditions (Irr), which appear in the blots from 01.06.2015 was ommitted in the blots from 03.06.2015 and as well in the main figure.
